# Antimicrobial Peptides: Their Role as Infection-Selective Tracers for Molecular Imaging

**DOI:** 10.1155/2014/867381

**Published:** 2014-08-27

**Authors:** Thomas Ebenhan, Olivier Gheysens, Hendrick Gert Kruger, Jan Rijn Zeevaart, Mike Machaba Sathekge

**Affiliations:** ^1^Department of Nuclear Medicine, University of Pretoria & Steve Biko Academic Hospital, Corner of Malherbe and Steve Biko Road, Pretoria 0001, South Africa; ^2^Catalysis and Peptide Research Unit, School of Health Sciences, University of KwaZulu Natal, E-Block 6th Floor, Westville Campus, University Road, Westville, Durban 3630, South Africa; ^3^Department of Nuclear Medicine, University Hospitals Leuven, Katholieke Universiteit Leuven, Campus Gasthuisberg, Herestraat 49, 3000 Leuven, Belgium; ^4^Department of Science and Technology, Preclinical Drug Development Platform, North West University, 11 Hoffman Street, Potchefstroom 2520, South Africa

## Abstract

Antimicrobial peptides (AMPs) are a heterogeneous class of compounds found in a variety of organisms including humans and, so far, hundreds of these structures have been isolated and characterised. They can be described as natural microbicide, selectively cytotoxic to bacteria, whilst showing minimal cytotoxicity towards the mammalian cells of the host organism. They act by their relatively strong electrostatic attraction to the negatively charged bacterial cells and a relatively weak interaction to the eukaryote host cells. The ability of these peptides to accumulate at sites of infection combined with the minimal host's cytotoxicity motivated for this review to highlight the role and the usefulness of AMPs for PET with emphasis on their mechanism of action and the different interactions with the bacterial cell. These details are key information for their selective properties. We also describe the strategy, design, and utilization of these peptides as potential radiopharmaceuticals as their combination with nuclear medicine modalities such as SPECT or PET would allow noninvasive whole-body examination for detection of occult infection causing, for example, fever of unknown origin.

## 1. Introduction 

Compared with other conventional technologies, tomographic imaging can evaluate disease processes deep within the body, noninvasively and relatively rapidly. It is therefore not surprising that molecular imaging has powerfully augmented the investigation of various disease processes and has become an essential tool in the field of oncology, for both research and patient care [1]. Another major advantage of imaging is its ability to provide a holistic, three-dimensional assessment of the whole organ or body, less likely to be limited by sampling errors and therefore corelating well with the overall disease process. While continued advances in molecular imaging have provided unparalleled opportunities for more refined methods to monitor diseases, tools for evaluating infection and inflammation remain limited. Two imaging methods, widely used in the clinics currently, include high resolution computed tomography (CT) that measures anatomic (and therefore late) changes or ^18^F-labeled 2-fluoro-deoxy-D-glucose (^18^F-FDG)-positron emission tomography (PET), which is a general marker of metabolic activity. As ^18^F-FDG is also accumulating in sites of infection and inflammation due to the elevated glucose metabolism in these loci [2], thus it is nonspecific for infection. Therefore it became increasingly important to develop more specific and selective infection imaging agents. Direct,* ex vivo*, labeling of leukocytes is considered the “gold standard” for infection imaging by PET. Unfortunately this process is very laborious and time-consuming and requires the handling of blood products [3–5]. Alternatively, indirectly labeled leukocytes can be achieved using radiolabeled molecules, such as chemotactic peptides or cytokines, that bind to receptors on the leukocytes [3]. Unfortunately the biological effects of some of the leukocyte receptor-targeting compounds have limited their clinical use as infection-specific molecular imaging agents [5]. Although the most commonly labeled leukocytes, neutrophils and lymphocytes, are quite selective for infection, there are cases when they may fail to detect an infection or accumulate in noninfected sites. If the infection fails to elicit an immune response, labeled leukocytes will not accumulate at the infected loci, which may be the case in a severely immune-compromised individual, or in the case of infection by certain pathogens, such as* Mycobacterium tuberculosis* or* Pneumocystis carinii*. Some noninfectious immune conditions, such as rheumatoid arthritis, may also provoke an immune response and accumulate the tracer [3]. Through the use of different tracers, different targeting strategies are possible to image infection using PET.

Tracers that interact directly with the pathogenic microbes responsible for infection are, by nature, highly specific for infection and unlike labeled leukocytes should not accumulate in sterile inflammations. These types of tracers include radiolabeled antibiotics and antimicrobial peptides. Technetium-99m labeled ciprofloxacin (^99m^Tc-ciprofloxacin) has been the most widely studied antibiotic-based tracer for SPECT infection imaging [6] targeting DNA Gyrase, an enzyme present in all dividing bacteria and is not thought to accumulate in dead bacteria or sterile inflammations. Some problems associated with its use as a tracer in SPECT infection imaging have occurred with regards to poor radiochemical purity and stability [3]. More recently it has been reported that localisation at infected foci takes place primarily through increased extravasation and stasis. This process also occurs at uninfected sites with increased vascular permeability and ^99m^Tc-ciprofloxacin may accumulate at sites of sterile inflammation thereby reducing its specificity for infection [7].

Antimicrobial peptides (AMP) have attracted interest as potential targeting vectors for the development of PET tracers designed for the detection of infection. These peptides are found in a variety of organisms including humans, and, so far, hundreds have been isolated and characterised. It is believed that these peptides function as broad-spectrum microbicides and form part of the innate immune system of many eukaryotes, including humans. Regardless of their origin, they share many common properties such as having a net positive charge, being amphipathic and, in most cases, are membrane active [8]. Due to their role in the body as a natural microbicide, these antimicrobial peptides are selectively cytotoxic to bacteria, whilst showing minimal cytotoxicity towards cells of the host organism. It is thought that the net cationic nature of the peptides results in a relatively strong electrostatic attraction to negatively charged bacterial cells and a relatively weak attraction to the eukaryote host cells, which are usually less negatively charged than prokaryotes, and is believed to form the basis of this cell-type discrimination [9]. The ability of these peptides to accumulate at sites of infection combined with their almost negligible cytotoxicity or attraction to host cells makes these peptides attractive as targeting vectors for PET imaging of infection [10].

## 2. Overview of Antimicrobial Peptides

Antimicrobial peptides are evolutionarily conserved biomolecules that form part of the defence mechanisms in many organisms [11], ranging from prokaryotes to multicellular animals such as humans [9]. They form part of the first line of defence against pathogenic microbes in higher animals and in many lower forms of life; they are the only form of defence against pathogenic and saprophytic microbes [12]. The selective cytotoxicity of these peptides, where they attack the pathogenic microbes and leave the host cells unharmed, is due to the fundamental differences in composition and structure of the host cells to those of the pathogenic bacteria and yeasts. Despite some AMPs showing immunomodulatory effects and/or chemotactic behaviour, a common feature of these antimicrobial peptides is that they are amphipathic but possess an overall positive charge [9]. Approximately 1500 antimicrobial peptides have been characterised in a wide range of organisms and classification of these peptides can be complicated due to the high degree of sequence dissimilarity between the various peptides. However, classification has been attempted based on amino acid composition and secondary structures.

Three large groups (Table 1) have been identified, namely, α-helical peptides, cysteine-containing *β*-sheet peptides, and flexible peptides rich in specific amino acids such as proline, tryptophan, histidine, arginine, and glycine [13].

### 2.1. α-Helical Antimicrobial Peptides

Approximately 30 to 50% of all antimicrobial peptides identified and studied to date contain predominant α-helical structures. This may be due the relative ease with which these peptides are chemically synthesised, which allows for extensive characterisation in the laboratory. These peptides usually consist of 12–40 amino acid residues and contain an abundance of helix stabilising residues such as alanine, leucine, and lysine but never cysteine. In aqueous solutions these peptides are often unstructured but assume their amphipathic α-helical conformations when associated with a cell membrane or in a membrane mimetic environment. Often these peptides are not strictly α-helices and may contain an internal kink [14].

### 2.2. *β*-Sheet Antimicrobial Peptides

The other major group of antimicrobial peptides are those that typically contain two to ten cysteine residues that form one to five interchain disulfide bonds. This bonding interaction allows these peptides to adopt the *β*-sheet conformation. Most *β*-sheet antimicrobial peptides are part of the defensin family and these peptides are evolutionarily conserved across plants, fungi, insects, molluscs, and vertebrate animals. Defensins typically consist of two to three antiparallel *β*-sheets stabilized by three to four intramolecular disulfide bonds; however in some cases an α-helical or unstructured segment is found at the N- or C-terminus. Unlike the α-helical antimicrobial peptides, which are unstructured in aqueous solutions, the defensins maintain a compact globular structure under such conditions [12, 13]. Apart from overall similarity in secondary structure, most mammalian-derived α-defensins possess two additional common features, namely, a protruding loop resulting from a conserved arginine/glutamate salt bridge and a *β*-bulge caused by a conserved glycine-X-cysteine (X: any amino acid) motif between the first and second cysteine residues [13].

### 2.3. Flexible Antimicrobial Peptides Rich in Specific Amino Acids

A minority of antimicrobial peptides contain a high proportion of certain amino acids such as proline, tryptophan, histidine, arginine, and glycine. Representative members of this class include the tryptophan rich bovine indolicidin and porcine tritrpticin, histidine rich human histatins, and the arginine and proline-rich porcine PR-39. Due to their unusual amino acid compositions, these peptides have highly variable secondary structures. The 13-amino acid indolicidin (ILPWKWPWWPWRR), for example, adopts a largely extended conformation in the presence of zwitterionic micelles composed of substances such as dodecyl-phosphocholine or anionic sodium-dodecyl sulfate [13].

## 3. Mechanisms of Cell Specificity and Selectivity of Antimicrobial Peptides

Inherent differences in the microbial versus the host cell membrane composition and architecture aid selectivity of the antimicrobial peptides. Regulation of expression or localisation of the peptides is also thought to prevent unwanted interactions with vulnerable host cells.

### 3.1. Target Specificity and Selective Cell Toxicity

**Figure 1:**
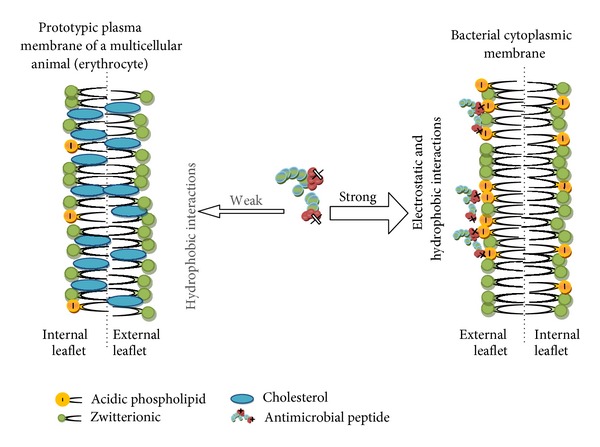
Membrane targeting of antimicrobial peptides and basis of their selectivity (adapted from [45]).

A biological membrane can be thought of as simply a fluid mosaic consisting of phospholipids interspersed with proteins. In different organisms glycerides and sterols may also contribute to the biochemical architecture and surface topology of such membranes. There are, however, fundamental differences that exist between microbial and animal cell membranes that allow the antimicrobial peptides to distinguish between these cells and selectively target one over the other as sketched in Figure 1 [9].

### 3.2. Membrane Composition, Charge, and Hydrophobicity

**Figure 2:**
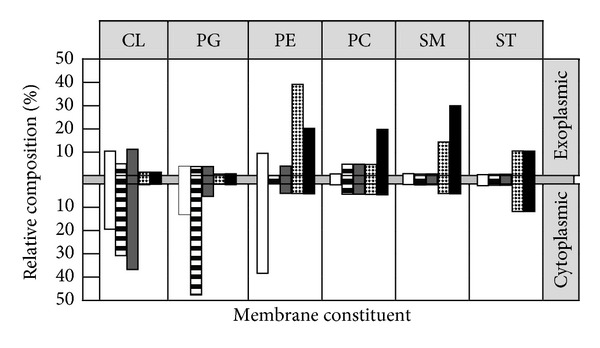
Comparative lipid architecture of microbial and human cytoplasmic membranes. Cytoplasmic membranes of bacterial (*Escherichia coli*,* Staphylococcus aureus*, or* Bacillus subtilis*) and fungal (*Candida albicans*) pathogens are compared with that of the human erythrocyte in relative composition and distribution between inner and outer membrane leaflets. Membrane constituents ranging from anionic (left) to neutral (right) are CL, PG, PE, PC, SM, and sterols (cholesterol or ergosterol, ST). Note the marked difference among microbial pathogens and human erythrocytes resides in the phospholipid composition and asymmetry. These differences are believed to account for the selective antimicrobial peptide affinity for microbial versus host cells to the extent that it exists for a given antimicrobial peptide. Keys: open,* E. coli*; horizontal hatching,* S. aureus*; shaded,* B. subtilis*; checkered,* C. albicans*; solid, human erythrocyte (adapted from [9]).

The core component of almost all natural biomembranes is the phospholipid bilayer. These bilayers are amphipathic, meaning they have both hydrophobic and hydrophilic regions. However, eukaryotic and prokaryotic cell membranes differ significantly in terms of exact composition and cell energetics (Figure 2). Phosphatidylcholine (PC) and its analogue sphingomyelin (SM) as well as phosphatidylethanolamine (PE) have no charge under physiological conditions [9]. Cholesterol and other sterols such as ergosterol which are abundantly found in eukaryotic membranes, but very seldom in prokaryotic membranes, are also generally neutrally charged (Figure 2) [15]. Hydroxylated phospholipids such as phosphatidylglycerol (PG), cardiolipin (CL), and phosphatidylserine (PS) possess a net negative charge under physiological conditions. It can be seen how the charge of the membrane is mainly due to the ratio and location of the various phospholipids, with cell membranes comprising mostly PG, CL, and PS, as is the case in most pathogenic bacteria, being very electronegative, whereas those membranes that are rich in PC, PE, or SP tend to have a net neutral charge, as is the case in mammalian cell membranes [15, 16].

### 3.3. Membrane Asymmetry

Although cellular membranes are neither symmetric nor static, differences between mammalian and microbial phospholipid bilayers can serve as potential targets for antimicrobial peptides. In some cells such as the bovine erythrocyte, only 2% of the total PE content is located on the outer membrane leaflet [13]. Differences in membrane symmetry, saturation of phospholipid bilayers, and compositional stoichiometry will influence the membrane's fluidity and phase transition. In a similar manner, the charge of the inner and outer leaflets of the cellular bilayer may also be different [15].

### 3.4. Microbial Ligands and Receptors as Targets for Antimicrobial Peptides

Experiments have shown that D-and L-amino acid versions of antimicrobial peptides exhibit similar binding affinities to targets cells, suggesting that stereospecific receptors are not involved in targeting pathogenic cells [9]. However, several studies appear to refute this and suggest that certain proteins located in the microbial cell membrane may serve as binding targets for certain classes of antimicrobial peptides such as histatins. This would support the findings why histadins are involved in local defence mechanisms with particular type of pathogens and have been recovered in dental or skin wounds. Some researchers also postulate that anionic components of cell membranes, for example, CL, PG, or lipopolysaccharide (LPS), may serve as pseudoreceptors, enabling the initial interaction between the antimicrobial peptide and the microbial cell target [13]. Hence, antimicrobial-binding receptors may be an alternative pathway of AMP interaction with the bacterial cell envelop.

### 3.5. Transmembrane Potential

The transmembrane potential is yet another way in which microbial and mammalian cells vary and it is in the charge separation that exists between the inner and outer layers of the cytoplasmic membrane. An electrochemical gradient, resulting from the differing rates or proton exchange across the cell membrane, is referred to as the transmembrane potential (Δ*ψ*). A normal mammalian cell has a Δ*ψ* between −90 and −110 mV in range. Pathogenic bacteria, however, generally exhibit Δ*ψ* in the −130 to −150 mV range. This significant difference in electrochemical potential may be another factor that allows antimicrobial peptides to distinguish between host and target cells [9].

## 4. Selective Toxicity Based on Antimicrobial Peptide Design

In the aqueous intercellular environment, many antimicrobial peptides are believed to adopt extended or unstructured conformations, although this may not be the case if there are intramolecular bonds present, which will ensure a specific conformation in a variety of environments due to induced rigidity. Once the antimicrobial peptide binds to the cell membrane of a pathogenic microbe, it may undergo significant conformational change and adopt a specific conformation, such as a α-helix. Studies suggest that dynamic and/or inherent conformations of antimicrobial peptides have an effect on their selective cytotoxicity [9, 17, 18]. Additionally, antimicrobial peptides may undergo conformational transition, self-association, or oligomerization within the target pathogen membrane, but not the host cell membrane to increase cell-specific toxicity [13]. Zhang and coworkers [16] employed synthetic test peptides that were uniformly cationic but varied in conformation and included extended, cyclic, α-helical, and *β*-sheet structures. It was determined that all test peptides were able to interact with and penetrate lipid monolayers composed of PG, a negatively charged phospholipid. However, only the α-helical and extended peptides were able to interact with the more neutrally charged PC membrane. In the same study it was also found that *β*-sheet peptides were able to translocate phospholipids from the inner to the outer leaflet at concentrations that were lower than those that were required to permeabilize the membrane. Similarly, Kol and coworkers [19] showed that peptides with comparable conformation, but rich in histidine and lysine and lacking in tryptophan, were also able to induce significant levels of phospholipid translocation. It can be concluded from these studies that not only do antimicrobial peptides interact with phospholipid membranes of only specific composition and symmetry, but they are also able to affect remodelling of the membranes in specific cells.

### 4.1. *In Vivo* Preferential Affinity for Microbial versus Mammalian Cells

Welling and colleagues [20] conducted an* in vivo* experiment where they tested the binding affinity of a radiolabeled fragment of the cationic ubiquicidin antimicrobial peptide ^99m^Tc-UBI 29–41 for microbial cells as compared to host cells. In the study, animals were infected with* Candida albicans*,* Klebsiella pneumonia,* or* Staphylococcus aureus*. Sterile inflammations were also induced in the thigh muscles of animals through injection of heat-killed microorganisms or purified LPS, to serve as controls. The radiolabeled peptides accumulated to a significant extent in the infected sites relative to sterile or noninfectiously inflamed parts of the body. This* in vivo* experiment demonstrated that the peptides could distinguish between host and microbial cells and also accumulate at the infected sites. Through scintigraphic measurements it was determined that the radiolabeled peptides accumulated in infected tissues at a rapid rate and that there was up to a fivefold increase in rates of accumulation in infected tissues relative to noninfected tissues. This rapid localization was interpreted as the peptides having a higher or preferential affinity for the target cell surface relative to that of the host cell surface.

### 4.2. Localisation of Cytotoxic Antimicrobial Peptides Limits Exposure of Vulnerable Host Tissues

It is possible that host cell cytotoxicity is reduced in many multicellular organisms due to their localization to tissues that are not vulnerable to their cytotoxic effects. In most animals these peptides are secreted by cells onto relatively inert and robust surfaces such as the epithelia of the intestines or lung, or in amphibians, onto the skin. These localities are most likely to interact with potentially harmful microbes most frequently, and the expression of most of the antimicrobial peptides is either constitutive or rapidly inducible, to allow them to form part of the first defences against pathogens [9]. Another means of protecting sensitive host tissues from antimicrobial peptides is by containing them within granules in the phagocyting leukocytes, which engulf pathogens and expose them to lethal concentrations of antimicrobial peptides and oxidizing agents. The defensin class of antimicrobial peptides is deployed in this way, since they are some of the most toxic and least selective of the host produced antimicrobial peptides. The slightly acidic microenvironment within the mature phagolysosome is also the most effective environment for the defensins, as they exhibit maximum cytotoxicity under these conditions [12].

## 5. Mechanisms of Antimicrobial Peptide Action

The generally conserved structures of antimicrobial peptides, across a wide variety of organisms, lend some clues as to their mechanisms of action. They are almost exclusively amphipathic and cationic under physiological conditions, and this is believed to aid their target cell selectivity. The ideal antimicrobial peptide should have low host cell cytotoxicity but be toxic to a wide range of pathogenic microbes. The antimicrobial determinants should be easily accessible and should not be prone to change or alteration. In general, antimicrobial peptides have amphipathic structures that allow them to interact with phospholipid membranes, structures that are essential to all pathogens [17]. Parameters such as conformation (*X*), hydrophobicity (*H*), hydrophobic moment (*M*_*H*_), charge (*Q*), polar angle (*θ*), and amphipathicity (*A*) are all important to the functioning of antimicrobial peptides. Furthermore, all these determinants are interrelated and modification of one of these features will lead to alteration of the others [9].

### 5.1. Conformation (*X*)

Although antimicrobial peptides may be found in a wide range of host organisms and have differing amino acid sequences, they can be classified into a few discrete groups based on their secondary structure. The two largest groups include peptides that possess a *β*-sheet or α-helical secondary structure. The majority of the remaining antimicrobial peptides are those that have an unusually high proportion of one or more amino acids such as tryptophan or proline and arginine. The α-helical peptides are frequently found in the intercellular fluid of insects and amphibians and generally adopt an unstructured or extended conformation in aqueous solution, only adopting their helical structure upon interaction with a phospholipid membrane [21]. The reason for this is that the intramolecular hydrogen bonding required for an α-helic conformation is disrupted in a polar solvent such as water. In a membrane, the polar hydrogen bonding groups are shielded from lipophilic (apolar) membrane environment through α-helic formation. The helix conformation also exposes the apolar side chains to the neutral lipid environment inside the membrane. Although the primary structure of the *β*-sheet class of antimicrobial peptides shows a level of dissimilarity in amino acid sequence, they all share common features with regard to amphipathic structure, possessing distinct hydrophilic and hydrophobic domains [9].

### 5.2. Charge (*Q*)

Most of the antimicrobial peptides are overall cationic and have charges ranging from +2 to +9, with many possessing highly defined negatively charged domains. This positive charge is important for the initial attraction to and interaction with the anionic cellular membranes of bacteria and other pathogenic microorganisms. Likewise the relatively less anionic membranes of the host do not electrostatically attract the antimicrobial peptides and may confer some target cell selectivity to the peptides. Pathogenic bacteria are generally rich in acidic phospholipids such as CL, PG, and PS. Additionally the teichoic and teichuronic acids of the cell walls of* Gram*-positive bacteria and the LPS of* Gram*-negative bacteria confer additional electronegative charge to the bacterial cell surface. It has been determined that the Δ*ψ* of bacteria is typically 50% higher than that of mammalian cells and it has been proposed that antimicrobial peptides may be concentrated onto the surface of pathogenic microbes in an electrophoretic manner [22]. Although many studies were able to correlate the cationicity of antimicrobial peptides with their antimicrobial activity, a strictly linear relationship does not exist. Dathe and coworkers [23] demonstrated in studies with analogues of magainin that increasing the cationicity from +3 to +5 resulted in an increase in antibacterial activity against both* Gram*-positive and* Gram*-negative species. They did, however, note that there was a limit to cationicity, after which any increases in positive charge no longer increase antibacterial activity. It is believed that this decrease in antibacterial activity may have been due to the peptides binding so strongly to the negatively charged phospholipid head group that translocation of the peptide into the cell was impossible [9].

### 5.3. Amphipathicity (*A*) and Hydrophobic Moment (*M*_*H*_)

**Figure 3:**
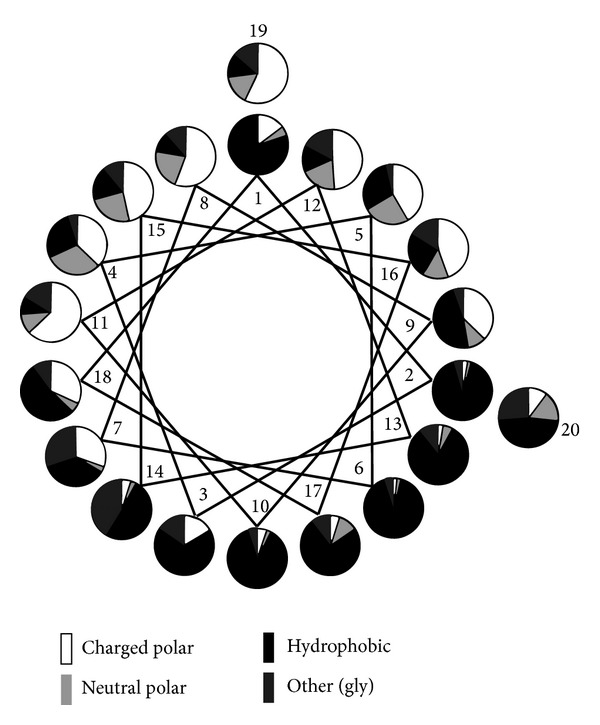
Statistical analysis of residue distribution in the 20-residue N-terminal stretch α-helical AMPs from natural sources. A graphical representation of the frequency of different types of residue at each position on a helical wheel projection is shown. The uneven distribution of hydrophobic and charged peptides contributes to the amphipathic nature of the peptide (adapted from Tossi et al. [17]).

Amphipathicity is a nearly universal feature amongst antimicrobial peptides and is achieved through a number of different peptide structures. The amphipathic α-helix is one of the most common and simplest of these features. By alternating anionic and cationic amino acid residues at every three to four positions the peptide is able to adopt a secondary structure that allows for optimal electrostatic interaction with amphipathic phospholipid membranes (Figure 3). This feature allows the peptide to exert cytotoxic activity towards not only negatively charged cell membranes but also those with a neutral charge or amphipathic nature [14].

Amphipathicity of a peptide can be described by its hydrophobic moment (*M*_*H*_) which can be calculated as the vectorial sum of individual amino acid hydrophobicities, normalized to an ideal helix. An increase in hydrophobic moment correlates to increased permeabilization of the target cell membrane. This is especially significant in interactions with lipid membranes that are neutrally charged, where charge factors are unlikely to bring about the required attraction to and interaction with the target cell membrane [17]. Like the α-helical antimicrobial peptides, the *β*-sheet host defence peptides also exhibit amphipathicity. This is manifested as varying numbers of *β*-strands organised to form hydrophobic and hydrophilic surfaces. The *β*-strands, which are often antiparallel, are stabilised by regularly spaced disulphide bonds or by cyclisation of the peptide backbone. This intramolecular bonding allows *β*-sheet antimicrobial peptides to maintain a rigid conformation even in aqueous extracellular fluid and also facilitates multimerization, as the hydrophobic surfaces will cluster together to avoid exposure to the aqueous environment. Although the exact mechanisms by which amphipathic antimicrobial peptides bring about membrane disruption in the target cell membrane is undetermined at present, largely because the exact conformation of the peptides in the membranes is not known, studies have shown that segregated amphipathicity in both α-helical and *β*-sheet antimicrobial peptides has a profound effect on peptide disruption of natural biomembranes [9].

### 5.4. Hydrophobicity (*H*)

The hydrophobicity of a peptide may be defined as the percentage of hydrophobic amino acid residues making up its primary structure. For most antimicrobial peptides the hydrophobicity is around 50% and is essential for the functioning of the peptide as it allows the peptide to interact with and penetrate into the phospholipid bilayer. Although a certain amount of hydrophobicity is essential for the functioning of the antimicrobial peptide, excessive hydrophobicity will increase its likelihood of destroying the host's cells and reduce its specificity for microbial cells [24]. Wieprecht and coworkers [25] studied the relationship between the hydrophobicity of peptides and their ability to permeabilize biomembranes. Using magainin analogues as model antimicrobial peptides, they were able to keep factors such as hydrophobic moment, helicity, and charge nearly constant, whilst producing analogues of variable hydrophobicity. Their experiments showed that hydrophobicity had little or no effect on the peptide's ability to bind to or permeabilize the membrane when it consisted exclusively of PG. However, in membranes consisting of a 3 : 1 ratio of PC : PG, the peptides with the highest hydrophobicity had an approximately 60-fold higher permeabilizing ability than the least hydrophobic peptide, and in membranes composed of only PC there was a 300-fold difference.

### 5.5. Polar Angle (*θ*)

A peptide's polar angle refers to the relative proportion of polar to nonpolar facets of the peptide conformed to an amphipathic helix. A helical peptide with one facet composed entirely of polar amino acid residues and the other facet composed entirely of nonpolar residues would have a polar angle of 180°. Less segregation between the domains, or an overabundance of hydrophobic residues, would lead to a lower polar angle. Studies conducted by Uematsu and Matsuzaki [26] on both synthetic and naturally occurring peptides have shown that a lower polar angle and therefore a more hydrophobic facet is more conducive to membrane permeabilization. Polar angle has also been correlated with the stability of peptide induced pores in biomembranes. They also demonstrated that antimicrobial peptides with smaller polar angles were able to induce higher degrees of membrane permeabilization and translocation at higher rates than peptides with greater polar angles. However, the pores formed by the peptides with smaller polar angles were less stable than those formed by peptides with greater polar angles. Hydrophobic and hydrophilic properties of antimicrobial peptides can be seen to play vital roles in the interactions with and permeabilization of phospholipid cell membranes [18].

### 5.6. Common Structural Features of Antimicrobial Peptides

Whilst a wide variety of antimicrobial peptides do exist in nature, conservation of key features and secondary structures has been noted. Extremes of features such as amphipathicity, charge, hydrophobic moment, or polar angle are not beneficial since they tend to compromise either antimicrobial activity or lead to increased host cell cytotoxicity. The minimum charge that peptides may possess in order to exert any kind of antimicrobial activity appears to be +2. This minimum cationicity is important because it allows for the initial electrostatic attraction to the bacterial membrane, which is negatively charged. It also allows for the displacement of any other cations that may already be bound to the target cell membrane and for the translocation into the interior of the membrane bilayer. Similarly, the hydrophobicity of the peptide should be moderate, since very hydrophobic antimicrobial peptides would target membranes with a net-neutral charge, such as the host cells, leading to a reduction in target selectivity and damage to the host organism. It can be seen that selective targeting of pathogenic microbes is largely due to a balance between electronegativity and hydrophobicity of the antimicrobial peptides [9].

## 6. **Initial Interactions with the Targeted Cellular Membrane**

The initial interaction between the antimicrobial peptide and the cell's phospholipid membrane is important as it determines target cell selectivity and also influences any subsequent interactions with the target cell. The initial interactions are largely determined by physical and chemical features of both the antimicrobial peptide and the target cell membrane [12].

### 6.1. Electrostatic Interactions

Electrostatic interactions are widely believed to be responsible for the initial targeting of the microbial cell. A study by Matsuzaki [27] correlated antimicrobial peptide cationicity with membrane binding ability, and the fact that cationicity is a conserved feature of almost all antimicrobial peptides in a wide range of organisms further supports this argument. Electrostatic forces act over a long range and the abundance of lysine and arginine residues in antimicrobial peptides, which are attracted to the negatively charged phosphate groups of biomembranes, lends further credibility to the theory that these interactions are responsible for the initial attraction to the target cell membrane [12]. In* Gram*-negative bacteria it is believed that the antimicrobial peptides displace cations that are normally associated with the LPS, since antimicrobial peptides possess a binding affinity for the LPS that is approximately three orders of magnitude greater than the divalent cations usually associated with this moiety. Strains of bacteria where the LPS is highly substituted with 4-amino-4-deoxy-L-arabinose or is highly acylated show greater resistance to positively charged antimicrobial peptides, lending further credibility to the theory that electrostatic charge is important for interaction with the target cell membrane [28].* Gram*-positive bacteria lack an LPS or outer cell membrane, but they do have a thick cell wall made up of teichuronic or teichoic acid polymers. These highly anionic structures are ideal targets for the cationic antimicrobial peptides. Strains of* Staphylococcus aureus* where the teichoic acids have been modified, resulting in increased anionic charge, are more susceptible to cationic antimicrobial peptides [29–31]. The fact that most bacteria have a strong electrochemical gradient (Δ*ψ*) relative to mammalian cells is also thought to increase target selectivity of antimicrobial peptides [9].

### 6.2. Receptor-Ligand Interactions with the Membrane

Some studies have shown that both naturally occurring and synthetic peptides interact with the membrane equally well regardless of whether D-amino or L-amino acids are used [32, 33]. This would suggest that interactions with biomembranes are not dependant on receptor-ligand mechanisms; however, other studies have shown that this may not be the case with all antimicrobial peptides. Nisin, a naturally occurring, cyclic peptide with powerful antimicrobial action has been found to bind specifically to bacterial membrane bound lipid II [34, 35]. Similarly, tachyplesin has been shown to have a specific affinity for LPS. The data from these studies suggests that receptor mediated binding is important for cell targeting in a small number of antimicrobial peptides [34].

## 7. **Events following Initial Membrane Binding**

Experimental determination of initial attraction of peptides to and interaction with cellular membranes is usually simpler than determination of interactions that follow this. A variety of methodologies such as circular dichroism [22, 36], X-ray crystallography, nuclear magnetic resonance [37], reverse phase-high performance liquid chromatography, and surface plasmon resonance [22], amongst other techniques, have been used to elucidate peptide-membrane interactions. However, it is suggested that the antimicrobial efficacy and mechanisms are extremely sensitive to conditions such as pH, osmotic strength, solution viscosity, and temperature, so any data obtained by the above mentioned techniques must be viewed with regard to these conditions [9]. Subsequent to the initial membrane binding, antimicrobial peptides penetrate the outer phospholipid membrane, a phase referred to as threshold concentration, and in doing so is able to exert their cytotoxic effects in the interior of the cell. The entry into the cell by the peptides requires a minimum number, or threshold concentration of antimicrobial peptides to accumulate on the surface of the lipid membrane. This event can be affected by factors other than concentration such as the ability of the peptides to multimerize and also features of the phospholipid membrane itself, such as its lipid composition, head group size, and fluidity [38]. The transmembrane potential of the bilayer may also influence the way in which the peptide enters the membrane, since a highly negative transmembrane potential will facilitate pore formation by drawing the positively charged peptide into the membrane [39].

## 8. Changes in Peptide Conformation upon Interaction with the Membrane

Many antimicrobial peptides, especially those with α-helical secondary structures undergo significant conformational rearrangement upon entering the nonpolar environment of the inner membrane. The α-helical antimicrobial peptides are normally disordered in the extracellular environment, exhibiting random coil or extended structures, but rapidly conform to a structured α-helix when associated with the biomembrane [18]. Some antimicrobial peptides can only undergo this conformational change in association with a negatively charged bilayer membrane. This may be due to the way the lipids are arranged in such membranes, with the phospholipid head groups inducing optimum periodicity of the cationic amino acid residues in the peptide, which in turn promotes correct conformation into the helical secondary structure [40, 41]. It has been suggested that this feature ensures that the antimicrobial peptides will only be “activated” into the cytotoxic form in the presence of the target cell membrane, in this case a negatively charged bacterium, and will not indiscriminately damage nontarget host cells [17]. The intramolecular disulphide bonds found in *β*-sheet peptides ensure that they maintain their secondary structure even in aqueous environments, and so they do not undergo the drastic conformational rearrangements seen in α-helical peptides, although quaternary peptide structures may disassociate upon entering the membrane, and this could facilitate selective toxicity [9]. Following the initial interaction with the cell membrane, many peptides may undergo self-association which, when combined with lipid-peptide interactions, may lead to the creation of complex structures that contribute to the cytotoxic effects of the peptide. The antimicrobial peptide's amino acid sequence and conformation in the monomer form will dictate its ability to form these structures. In amphipathic peptides, the hydrophobic domains are able to interact with the nonpolar hydrophobic core region of the lipid bilayer thereby driving the peptide deeper into the membrane. Alternatively they could also interact with the hydrophobic facets of other peptides, promoting multimerization in an attempt to avoid exposure of these facets to the aqueous environment. This type of multimerization and interaction with the interior of the lipid bilayer may result in peptide lined pores or channels being formed in the biomembrane, resulting in loss of integrity and permeabilization. Since biomembranes are highly variable in composition and structure, it is possible that a peptide may behave in a number of different ways when associated with different cellular membranes [9]. Several models have been proposed to describe the pore formation observed in membranes that have been exposed to antimicrobial peptides.

### 8.1. The Barrel-Stave Model

**Figure 4:**
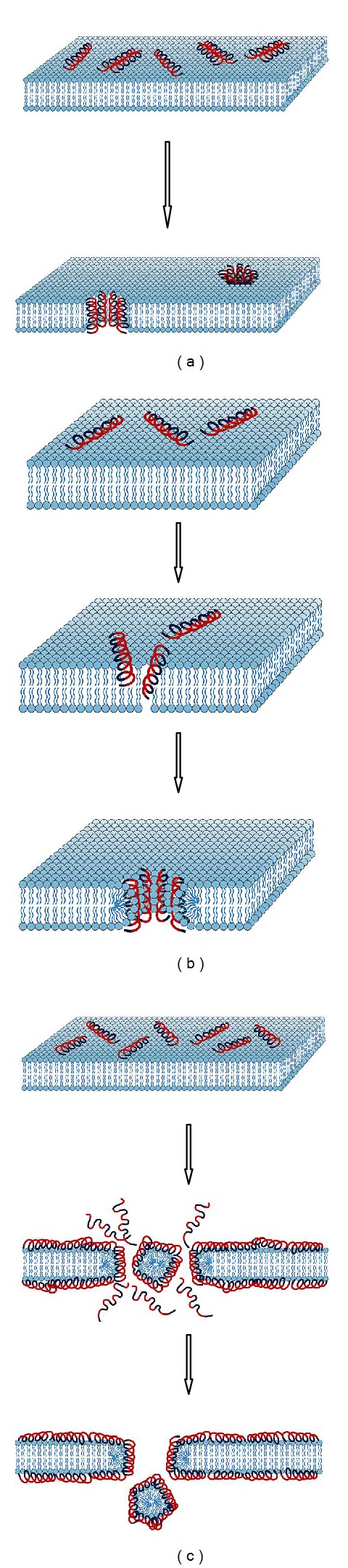
Overview of possible interaction mechanism following peptide interaction with the bacterial cell membrane [53], that is, (a) barrel-stave model (pore formation), (b) toroidal model (pore formation), and (c) carpet model (membrane disruption). Red coloured peptide regions: hydrophilic; blue coloured peptide regions: hydrophobic.

This mechanism of membrane pore formation is so named because the transmembrane peptides, or peptide complexes, lining the channel are positioned in a barrel-like ring, with the peptides forming transmembrane staves. Amphipathic peptides are oriented so that the hydrophobic domains interact with the nonpolar hydrocarbon tails located in the interior of the lipid membrane, whereas the hydrophilic domains are oriented so that they face the aqueous channel of the pore and form its lining [24]. Initially the monomer peptides accumulate at the cell surface and undergo conformational rearrangement when they contact the membrane (Figure 4). This is thought to force the phospholipid head groups aside and induce thinning of the membrane. This allows the hydrophobic part of the peptide to enter into the nonpolar interior of the membrane, whilst the cationic amino acids of the antimicrobial peptide interact with the negatively charged head groups. When the threshold concentration of the peptides is reached the peptide monomers are able to aggregate to form multimers which further forces the peptides into the hydrophobic centre of the membrane, as the aggregation prevents the hydrophilic parts of the peptide from being exposed to the hydrophobic parts of the inner membrane (Figure 4(a)). As ever increasing numbers of peptide monomers aggregate, the pore in the membrane is expanded [9, 24].

### 8.2. The Toroidal Pore or Worm-Hole Mechanism

This mechanism of pore formation has been well studied using the α-helical magainin peptides. Upon contacting the charged cellular membrane, the disorganised peptides take on the α-helical structure. Initially the helices orientate themselves so that they are parallel with the surface of the membrane. The polar phospholipid head groups are displaced and the surface of the membrane is weakened, resulting in a positive curvature strain in the membrane. As a result of this strain and thinning, the membrane is destabilised and becomes more susceptible to further peptide interactions. Once a threshold concentration of peptides is reached the peptides reorientate so that they are perpendicular to the membrane and begin to multimerize so that the hydrophilic parts of the peptides are not in contact with the hydrophobic parts of the membrane (Figure 4(b)). The newly formed toroidal pore is unstable and upon disintegration some of the peptides are forced into the inner leaflet of the cell membrane. It is therefore believed that the disintegration step of these transient pores is important as it allows the peptides to translocate into the intracellular space, where they may act on other targets [42].

### 8.3. The Carpet Model

The carpet model of membrane permeabilization is based on diffuse action by many monomer peptides on the cellular membrane. When sufficiently high concentrations of certain antimicrobial peptides are present on the cell membrane some of the phospholipids of the membrane are displaced which results in changes to the membranes fluidity or brings about weaknesses in the barrier properties of the membrane. The cumulative effect of these displacements is that the membrane is weakened and loses its integrity. As suggested previously, the initial attraction of the antimicrobial peptides to the membrane is through electrostatic attraction forces. No specific channels or pores are formed, and it is believed that permeabilization and loss of membrane integrity are through the unfavourable energetic properties that dispersion of the phospholipids brings about (Figure 4(c)) [22].

## 9. Impact of Bacterial Infections to Human Health and Traditional Methods of Infection Diagnosis

It is estimated that up to 85% of patients that are critically ill in hospital have a fever but display no other outward sign of infection. Since prolonged episodes of fever can be fatal, it is essential that any underlying infection is detected as soon as possible, so that the correct treatment regime can be initiated [43]. Traditional methods of diagnosis may include examination of tissue biopsies and attempting to culture pathogens, an often inaccurate and time-consuming task which can delay the onset of treatment. Diagnostic imaging procedures are also employed and may include computed tomography (CT) scanning or magnetic resonance imaging (MRI). However, these techniques are generally not able to detect early stage infections as they require morphological changes in the tissues to take place, a feature usually associated with advanced infections [44]. Furthermore, they are generally focussed on specific parts of the body, meaning that it is possible that the infection may be missed, or the true extent of the infection may not be detected. Gallium-radiolabeled antibodies or -immunoglobulins or complexes such as ^67/68^Ga-citrate may be employed to highlight regions where leukocyte trafficking is occurring using SPECT or PET scanning. However, these technologies are unable to definitively distinguish between infected tissues and those that are inflamed but sterile, since leukocyte trafficking occurs in both cases [44]. Given the high specific affinity of naturally occurring antimicrobial peptides for pathogenic bacteria or fungi, as opposed to cells of the host organism, it was envisaged that they may be employed to aid the resolution of diagnostic imaging processes [45].

### 9.1. The Use of Antimicrobial Peptides as Radiopharmaceuticals

Ideally, a radiopharmaceutical employed for infection imaging should allow for rapid detection of bacteria and rapid clearance from the noninfected sites. It should also exhibit high and specific uptake at the infected site, with minimal amounts accumulating in sterile or nontarget tissue. The compound should also have low toxicity and not induce an immune response. Very importantly, it should be able to distinguish between a sterile and an infected inflammation [45]. Since antimicrobial peptides generally show a broad spectrum of activity against a wide range of pathogenic yeasts and bacteria they are ideal targeting molecules for infections where the suspected pathogen has not been identified. Additionally their mode of action requires them to physically associate with the pathogen, and so they would be able to bring a gamma or positron emitting source, such as technetium-99m (99mTc) or gallium-67 (^67^Ga), to the exact location of the infection. Their lack of affinity for the host organism's cells also means that they would not accumulate in sterile inflamed tissues. Radiolabeled antimicrobial peptides are also attractive because they are cleared rapidly from the circulatory system and excreted by the body. In addition they are also able to penetrate the extravascular tissues and thereby accumulate at infected sites in a very short space of time [46]. Ideally, the radiolabeling procedure of a targeting molecule should allow for the firm attachment of a radionuclide to the molecule without it adversely affecting its targeting ability or the pharmacokinetics of the molecule. Labelling approaches can either be direct or indirect as follows.

**Figure 5:**
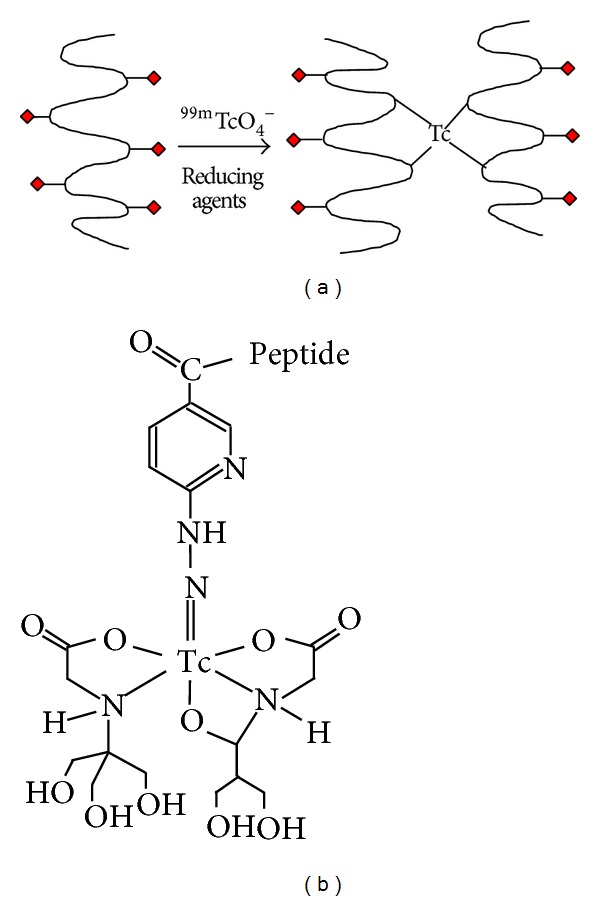
Approaches in radiolabeling of peptides. The direct method (a) where radionuclides are covalently attached to the peptide and the indirect method (b) where radionuclides are attached to targeting peptides by means of bifunctional chelators [8].

- A direct labelling (Figure 5(a)) approach involves incorporation of the radionuclide onto the targeting molecule* via* a covalent bond. In the case of peptide targeting molecules a covalent bond may be formed between the radionuclide and a suitable free amide residue of Lys and Arg [47]. Using the tyrosine residue may cause problems associated with labelling including nonspecific or poor binding,* in vivo* instability of the complex, and unwanted alterations to the peptide structure, such as the cleaving of internal disulphide bonds, which can alter its functioning [8].
- An indirect labelling strategy can be used through addition of chelating agents to the targeting molecule (Figure 5(b)) [8]. Bifunctional chelates have been used to label peptide carrier molecules with radionuclides. The chelating agent may be preloaded with the radionuclide prior to being bonded to the carrier moiety, or it could be firstly attached to the carrier molecule and then exposed to the nuclide for chelation in a process known as postlabelling. Postlabelling has the advantage that the carrier molecule can be stored for a long period of time until needed, and the radionuclide, which undergoes decay, can be added shortly before the radiopharmaceutical is administered. This benefits commercialisation of the carrier molecule and makes the technology easier to use in hospitals or clinics [48].

### 9.2. Ubiquicidin Exemplifies an Approach for Antimicrobial Peptide Derived Radiopharmaceuticals

**Figure 6:**
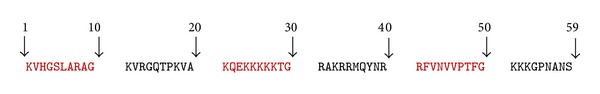
Primary structure of ubiquicidin as originally reported by Hiemstra and coworkers [49].

The 59-amino acid residue antimicrobial peptide ubiquicidin (UBI) is a 6.7 kDa peptide that was first discovered in cytosolic extracts of the murine macrophage (Figure 6). This peptide was shown to exhibit antimicrobial effects against* Salmonella typhimurium* and* Listeria monocytogenes*. It was subsequently found in a wide range of other organisms, including humans [49]. Since it occurs naturally in man, ubiquicidin is not an immunogenic entity, which makes it suitable for administration as a diagnostic tool. It also has high affinity for bacterial cells but does not target mammalian cells, rendering it nontoxic to the patient and selective in that it is unlikely to accumulate at sterile inflammation sites [50]. Several studies have been performed on fragments of ubiquicidin both* in vitro* and* in vivo* to assess its ability to bind to bacterial cells.

Welling and coworkers [51] evaluated the whole ^99m^Tc labeled ubiquicidin and various radiolabeled fragments of the peptide, including UBI1-18 (KVHGSLARAGKVRGQTPK), UBI29-41 (TGRAKRRMQYNRR), UBI18-29 (KVAKQEKKKKKT), UBI 18–35 (KVAKQEKKKKKTGRAKRR), UBI31-38 (RAKRRMQY), and UBI22-35 (QEKKKKKTGRAKRR) for their ability to bind to bacterial cells and/or human leukocytes* in vitro*. They found that the ubiquicidin peptide fragments UBI 18–35, UBI 31–38, UBI 22–35, and UBI 29–41 showed considerably higher binding affinities for the bacterial cells than they did for the human leukocytes. The* in vivo* results, obtained by scintigraphy of experimentally infected mice following intravenous administration of the various radiolabeled peptides showed that the UBI18-35 and UBI29-41 peptides appeared to be the most promising candidates. After a postadministration period of 2 h and 24 h, the leukocyte to bacteria binding ratios were 1 : 36, 1 : 166, and 1 : 73, 1 : 220 for UBI18-35 and UBI29-41, respectively. The researchers concluded that UBI29-41 and UBI18-35 were the optimal peptides for distinguishing infections from sterile inflammations.

### 9.3. Human Clinical Trials of ^99m^Tc-Ubiquicidin 29–41 as an Infection Imaging Agent

**Figure 7:**
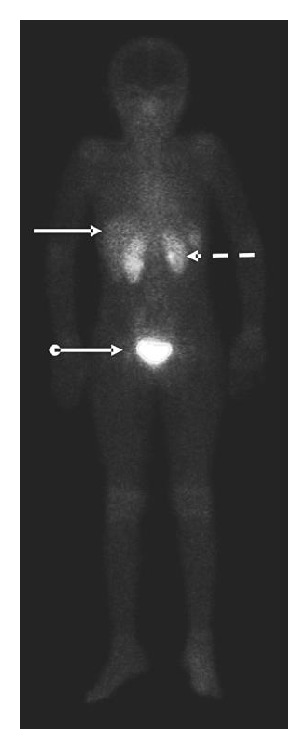
Anterior whole-body image taken at 30 min after tracer injection showing kidneys (dotted arrow), liver (solid arrow), and urinary bladder (ball arrow) (adapted from [52]).

Akhtar and coworkers [52] studied the efficacy of ^99m^Tc-UBI 29–41 as an infection imaging agent in eighteen patients with suspected prosthetic or soft tissue infections. Using scintigraphy to monitor the radiolabeled peptide, the researchers were able to monitor the target to nontarget (T/NT) ratios of the imaging agent. Infection in the patients was confirmed through culture of bacteria from the infected site, or where this was not possible through complete blood examination. The study found that all patients tolerated the radiolabeled peptide well, no significant changes to their vital signs were noted, and no related side effects were seen following the administration of the ^99m^Tc-UBI 29–41. The T/NT ratio was determined at 30, 60, and 120 minutes, with the 30-minute scan showing the highest mean T/NT value. The anterior whole-body scan (Figure 7) gave information about the biodistribution of the tracer and its routes of elimination by the body. It can be seen that the tracer is mostly eliminated by the urinary system and also some perfusion-dependant liver activity was noted. The imaging agent was found to have a sensitivity of 100% and a specificity of 80%. The researchers concluded that the ^99m^Tc-UBI 29–41 had a positive predictive value of 92.9%, a negative predictive value of 100%, and an overall diagnostic accuracy of 94.4%. The radiolabeled peptide displayed efficacy against a range of different bacteria, including* Pseudomonas aeruginosa*,* Staphylococcus aureus,* and* Streptococcus pyogenes*. It was the opinion of the researchers that ^99m^Tc-UBI 29–41 is a highly sensitive and specific imaging agent for detecting soft tissue and bone infections in humans.

## 10. Discussion and Perspective

The usage of nuclear medicine modalities such as SPECT or PET allows clinicians for noninvasive whole-body examination of physiological processes such as occult infection at cellular level and, apart from being a useful tool for physiological and medical research, these highly sensitive technologies are capable of detection of diseases without, or prior to, anatomical change (fever of unknown origin). To date, radiolabeled leukocytes, monoclonal antibodies against cytokines/leukocytes, and tracers associated with specific molecular targets or metabolic processes are utilized [54]. Radiolabeled leukocytes have a spectrum of limitations (alteration of leukocyte function due to radiation damage), that is, they have a cumbersome pharmakokinetic and are also relatively nonspecific. Moreover, labeled leukocytes and high molecular weight tracers such as antibodies may also have limited penetration into infected or diseased tissues. The latter presented overview which clarifies the widespread potential of AMPs to be evaluated as imaging probes, given their unique selective involvement with bacteria. A simple literature query, searching for “antimicrobial peptides” resulted in ca. 6000 publications. However, as soon as the query is combined with the term “imaging”, it resulted in only 63 publications; only 17 of those have clinical relevance (^99m^Tc-UBI-29-41 related studies/trials). This is an important observation as this ubiquicidin fragment may represents a near-perfect carrier for targeting molecules for infection detection. The human clinical trials conducted by Akhtar and coworkers [52] with ^99m^Tc-UBI 29–41 did not find any evidence of cytotoxicity in the patients which supports the findings of the current study. Even though it was stated that the signal to noise ratio is low [46], it has been used successfully for 10 years now. In 2010 the clinical trials to date were justified by de Murphy et al. towards their diagnostic value over the initial 7-year period. ^99m^Tc-UBI 29–41 meta-analysis returned high values for sensitivity (96.3%), specificity (94.1%), and accuracy (95.3%) with high positive predictive (95.1%) and negative predictive values (95.5%) [54]. From 2011 onwards, seven additional clinical studies (enrolling together over 160 patients) have been successfully carried out and all demonstrated ^99m^Tc-UBI29-41-SPECT as a highly accurate and selective diagnostic tool for bone infection in diabetic foot [55], hip prostheses [56], or other implant related infections [57, 58]; moreover it also detects osteomyelitis [59, 60] and infective endocarditis [61]. It can be ascertained that this field of applications for ^99m^Tc-UBI29-41 imaging will keep growing also because research with alternative radioisotopes other than ^99m^Tc may yield a new group of radiopharmaceutical agents for medical diagnostic imaging using clinical PET/CT or PET/MRI in future. Novel radioisotopes such as ^68^Ga, ^82^Rb, or ^62^Cu can be produced on-demand from a radioisotope generator without the need for an on-site cyclotron and may serve as radionuclides for PET. ^68^Ga has garnered interest as a positron emitter for molecular imaging due to some of the advantages it offers in a tracer. It has a radioactive half-life of 67.71 minutes which makes it compatible with biokinetics of most low molecular weight radiopharmaceuticals such as peptides, oligonucleotides, aptamers, or antibody fragments. The nuclear decay of the isotope is mainly through positron emission (89%), with average positron energy of 740 keV. Additionally, the coordination chemistry of Ga^3+^ is well understood, which is helpful in designing chelating agents which can be used to link this radionuclide to a targeting vector [62]. Recently, UBI29-41 was conjugated to the macrocycle 1,4,7-triazacyclononane-1,4,7-triacetic acid (NOTA) and subsequently labeled with ^68^Ga [63]. This approach was initially utilized with 1,4,7,10-tetraazacyclododecane-N′,N′′,N′′′,N′′′′-tetraacetic acid (DOTA) to yield the peptide derivatives such as DOTA-TOC or DOTA-TATE for ^68^Ga-complexation which subsequently allowed tumor-receptor-based PET imaging. In a preclinical study using ^68^Ga-NOTAUBI29-41-PET it was shown that the macrocycle conjugation did not compromise the peptide's ability to selectively bind to bacteria* in vivo* [64]. Aside from UBI, there are other compounds evaluated for infection and inflammation imaging [54] but the majority of antimicrobial peptides available remain underinvestigated in terms of infection imaging. In 2000 the human neutrophil peptides (HNP1-3) were considered amongst other peptides as useful agent for targeting infection, as part of the defence mechanism in monocyte/lymphocyte cultures HNPs plays a chemotactic role as mediating molecules. This ambiguous role may be a drawback in developing HNPs for imaging; hence the usage of particular peptides as targeting vectors may have some secondary limitations, despite of their favourable cellular properties [65]. As radiopharmaceuticals are mostly administered by i.v. injection, the peptides can be prone to enzymatic degradation or the destabilisation of the radioisotope as reported for ^18^F-UBI29-41 [66]. The lactoferrin-derived peptide hLF(1-11) showed great sensitivity as infection agent targeting multidrug-resistant* Acinetobacter baumannii* strains; however binding to* Candida albicans*, a fungus, and the hepatobiliar excretion made it less favourable for imaging [51]. Moreover hLF showed immune-activating or bactericidal effects depending on the dose administered, that is, encountered with a negative feedback mechanism by interleukin-10 modulation [67, 68]. Another example, the AMP Latarcin-2a, extracted from the venom of the Central Asian spider* Lachesana tarabaevi*, has undesirable lytic activity against Gram-positive and Gram-negative bacteria, erythrocytes, and yeast at micromolar concentrations and thus makes it less considered for bacterial detection with PET [69]. Moreover, most bacteria are able to produce both, surface-bound and/or secretory proteases, a defence strategy that can degrade or inactivate AMPs. Consequently, using AMP-derived compounds as imaging agents would result in false-negative diagnostics where a persistent infection might easily be misjudged or overseen entirely. Through understanding these specific bacteria-intrinsic defence mechanisms, it can be avoided to use vulnerable AMP-derived structures as infection imaging agents. It should also be noted that, except for a few structures, research did not reveal a bacteria-specific receptor-like target that complements the potential peptides as a ligands or allosteric modulators. Tumor cells, in contrast, express specific receptors integrin-, bombesin-, or somatostatin ligands or antagonists which are targeted by SPECT or PET tracers [10, 70]. Moreover, the host's immune system, when reacting on infections, has pathologic pathways that can be imaged using PET. Activated macrophages may act as an equivalent host-dependent target which can be visualized by ^18^F-FDG nonspecifically [3] but the actual bacterial burden remains overseen. In contrast, AMP-derived peptides are acting in a host-independent mechanism: radiolabeled peptides will bind to free and to cell-adherent but not phagocytised bacteria and hence bacteria become invisible for ^99m^Tc-UBI29–41-SPECT once they are incorporated by macrophages [71]. The use of this modality potentially allows for the early detection of infection prior to any morphological changes in the body taking place [7]. It also allows for the discrimination of infection from a sterile inflammation which may appear superficially similar as both may present as reddened, swollen, and unusually warm areas. This is due to the increased blood flow, enhanced vascular permeability, and influx of white blood cells which are common in both situations [4]. The latter approach would emphasize a dual tracer imaging regimen in future clinical studies or even dual tracer administration (if the respective radioisotope properties and pharmacokinetic properties are complementing the approach). In summary, the ideal tracer for clinical PET imaging of infection should meet several criteria. (1) It should sustain substantial blood degradation and have a reasonable degree of lipophilicity; (2) it should accumulate and be retained at the site of infection (ideally by internalization and subsequent amplification), with minimal accumulation in noninfected sites; (3) it should have rapid clearance of nonspecific activity uptake from the surrounding regions for high signal-to-noise-ratios; and (4) it should have minimal side effects and should be easy to prepare, at low cost. UBI29-41 has proven its usefulness towards generic infection imaging, and other suitable AMPs based radiopharmaceuticals will follow, undoubtedly.

## Figures and Tables

**Table 1:** Representative antimicrobial peptides of different classifications (modified from [13]).

Class	Representatives	Host
*α*-helical	LL-37	Mammal: human
	Cecropins	Insect: moth
	Melittin	Insect: honey bee
	Magainins	Amphibian: frog
	Fowlicidins	Ave: chicken

*β*-sheet	Thanatin	Insect: soldier bug
	Tachyplesins	Arthropod: horseshoe crab
	Protegrins	Mammal: pig
	Plant defensin VrD2	Plant: mung bean
	Plectasin	Fungus: ebony cup
	Insect defensin A	Insect: northern blow fly
	α-defensin	Mammal: human
	*β*-defensin	Mammal: human
	*θ*-defensin	Mammal: rhesus monkey

Flexible	Indolicidin	Mammal: cow
	Tritrpticin	Mammal: pig
	Histatins	Mammal: human
	PR-39	Mammal: pig

## Abbreviations

AMP: Antimicrobial peptides
*B. subtilis*: * Bacillus subtilis*
*C. albicans*: * Candida albicans*
CL: Cardiolipin
CT: Computed tomography
DNA: Deoxyribonucleic acid
*E. coli*: * Escherichia coli*
FDG: Fluorodeoxyglucose
LPS: Lipopolysaccharide
MRI: Magnetic resonance imaging
PC: Phosphatidylcholine
PE: Phosphatidyl-ethanolamine
PET: Positron emission tomography
PG: Phosphatidyl-glycerol
PS: Phosphatidyl-serine
*S. aureus*: * Staphylococcus aureus*
SM: * Sphingomyelin*
SPECT: Single photon emission computed tomography
ST: Sterols
T/NT: Target to nontarget ratio
TATE: (Tyrosine^3^)octreotate
TOC: (Phenylalanin^1^-Tyrosine^3^)octreotide
UBI: Ubiquicidin (fragment).
